# Regional differences in Acute Kidney Injury incidence and mortality in developing countries: recent trends

**DOI:** 10.1590/2175-8239-JBN-2020-0150

**Published:** 2020-09-11

**Authors:** Etienne Macedo, Ravindra L. Mehta

**Affiliations:** 1 University of California, Division of Nephrology and Hypertension, Department of Medicine, San Diego, CA, USA.

Acute Kidney Injury (AKI) is a global public health problem with short and long-term consequences that affect patients’ quality of life^1^^-^ 5. The widespread availability of electronic health records in developed countries has improved our understanding of the epidemiology of AKI incidence, rates of recovery, and progression. However, in the developing world, lack of electronic health data has limited comprehensive assessments of the burden of AKI. It is well recognized that the burden of AKI is often underrepresented in the developing world and has prompted initiatives to improve awareness of AKI and develop strategies for its prevention and management^6^. Previous studies have shown different etiologies and outcomes following AKI in developed and developing countries based on governmental health service infrastructure and expenditures^7^. In a recent metanalysis, patients’ characteristics were mostly similar in high and low and low middle-income countries, but outcomes were worse for patients in developing countries^7^. Nevertheless, very heterogeneous outcomes can be found within the same country as regional differences in the availability of health care resources and access to care influence patient management.

The study by Herrera-Añazco et al.^9^ in this issue of the Brazilian Journal of Nephrology describes regional trends in age-standardized incidence and mortality rates of AKI in Peru. They evaluated AKI incidence, based on ICD coding, from the Ministry of Health (MINSA) database and correlated with mortality statistics based on death certificates issued by the National Registry of Identification and Civil Status of Peru (RENIEC) over two different periods, between 2005-2010 and 2011-2016. They found an increase of AKI incidence per 100,000 population and a decrease in mortality rates in the later period. There was a considerable variation on the incremental frequency; from negative 56% change to positive 542% in different regions. Interestingly, the Tumbes region with the greatest increase in the incidence of AKI also had the greatest reduction in mortality; however, this was not seen in other regions. Whether regional differences across the two time periods represent improved recognition, better access to care, or better coding for AKI is not clear. Nevertheless, the data raise interesting questions for health policy considerations. As stated by the authors, underreporting can be a major issue when using public health data from the ministry of Health as private hospitals were not included in the analysis. In addition, less severe cases are often not recorded for AKI diagnosis. In the poorest regions, limited access to healthcare services, limitations in the diagnosis are also associated with underreporting. These issues suggest that the true incidence of AKI in Peru may be somewhat higher than it appears to be in this study for age-adjusted rates and should prompt additional studies to delineate the prevalence of AKI and its consequences.

The location of AKI development, if hospital-acquired or community-acquired, also needs to be considered as community-acquired AKI is often not detected if patients are not admitted to a health care facility. Since AKI is not associated with any specific symptoms, and the diagnosis is mostly based on the measurement of lab parameters, AKI is often not recognized. The etiologic spectrum of hospital-acquired AKI in developing countries, which has been described primarily in large urban centers in these nations, is similar to the causes in more developed countries. It includes post-surgical complications, hemorrhage, infections, septic shock, and drug toxicity. In contrast, community-acquired AKI in developing counties is mostly encountered in rural areas. Its true prevalence and leading causes are not well known as a consequence of limited diagnostic capacity and lack of awareness by health care workers. Shortage of infrastructure, human resources, and challenges on accessing health are associated with worse outcomes, with some studies showing mortality rates can reach levels up to 80%^2^^,^ 9 when renal replacement therapy (RRT) is necessary.

The different AKI definitions used over time can affect the comparison of incidence over the studied years. Studies based on administrative data relying on ICD coding need to consider the current definitions for AKI were introduced in 2007 and represented in the ICD coding some years later. Additionally, details of the diagnostic criteria used for reference serum creatinine, oliguria, and the timeframe for AKI assessment are not available in administrative data. Accurate information on AKI can help to drive resources that ultimately can improve outcomes. In developed countries, the easy access to patients’ data, laboratory exams, and computerized systems have improved the quality of information on AKI incidence and etiological factors. The knowledge that even mild AKI cases are associated with increasing mortality and the effects of an AKI episode on long-term outcomes has driven more investment into early detection and treatment of AKI.

The discrepancy in the mortality rate showed by Herrera-Añazco et al.^8^ among the different regions is also remarkable, varying from an increment of 226 % change to a decrement of minus 69%. However, it is difficult to determine whether the mortality differences can be attributed to AKI, given the heterogeneity of underlying comorbidities, related conditions during hospitalization, and process of care. As pointed by the authors, in developing countries, limited availability of health workers, diagnostic equipment, and limited hospital resources are among the reasons related to poor AKI outcomes. Rural areas can lack the infrastructure for AKI treatment, and a structured referral system is crucial in order to optimize AKI management. In more severe AKI cases, limited availability of trained personnel and RRT equipment can limit treatment options.

In developed countries, it is increasingly recognized that a substantial proportion of the total AKI burden is due to incomplete renal recovery with subsequent chronic kidney disease. Inadequate follow-up of kidney function is an even more significant problem in developing countries, affecting patients’ chance of recovery and progression to CKD. Patients surviving an AKI episode are rarely followed by a nephrologist^10^. Ideally, patients should be monitored for more than three months after discharge and have their SCr level routinely measured to assess renal function and renal injury progression. With the increasing number of patients recovering from AKI, it is necessary to direct efforts into education and health providers’ training to follow kidney function recovery. Appropriate management of patients with incomplete kidney recovery may delay the progressive loss of kidney function, ultimately preventing the incremental increase in chronic dialysis need. Educational campaigns on the importance of long-term follow up of AKI patients must be planned according to the level of health organization and involve the whole health care team, including physicians, nurses, and medical allied personal.

## References

[1] Magro MCS, Vattimo MFF (2007). Avaliação da função renal: creatinina e outros biomarcadores. Rev Bras Ter Intensiva.

[2] Schrier RW, Wang W, Poole B, Mitra A (2004). Acute renal failure: definitions, diagnosis, pathogenesis, and therapy. J Clin Invest.

[3] Vieira JM, Castro I, Curvello-Neto A, Demarzo S, Caruso P, Pastore L (2007). Effect of acute kidney injury on weaning from mechanical ventilation in critically ill patients. Crit Care Med.

[4] Bouchard J, Soroko SB, Chertow GM, Himmelfarb J, Ikizler TA, Paganini EP (2009). Fluid accumulation, survival and recovery of kidney function in critically ill patients with acute kidney injury. Kidney Int.

[5] Fischer MJ, Brimhall BB, Lezotte DC, Glazner JE, Parikh CR (2005). Uncomplicated acute renal failure and hospital resource utilization: a retrospective multicenter analysis. Am J Kidney Dis.

[6] Mehta RL, Burdmann EA, Cerdá J, Feehally J, Finkelstein F, García-García G (2016). Recognition and management of acute kidney injury in the International Society of Nephrology 0by25 Global Snapshot: a multinational cross-sectional study. Lancet.

[7] Melo FAF, Macedo E, Fonseca Bezerra AC, Melo WAL, Mehta RL, Burdmann EA (2020). A systematic review and meta-analysis of acute kidney injury in the intensive care units of developed and developing countries. PLoS One.

[8] Herrera-Añazco P, Ccorahua-Ríos MS, Condori-Huaraka M, Huamanvilca-Yepez Y, Amaya E, Atamari-Anahui N (2020). National trends in age-standardized incidence and mortality rates of acute kidney injury in Peru. J Bras Nefrol..

[9] Bellomo R, Kellum JA, Ronco C (2004). Defining acute renal failure: physiological principles. Intensive Care Med.

[10] United States Renal Data System (USRDS), National Institutes of Health (NIH) (2007). 2007 Annual report - Atlas of chronic kidney disease and end-stage renal disease in the United States. Maryland,.

